# Pathway-Based Analysis Revealed the Role of Keap1-Nrf2 Pathway and PI3K-Akt Pathway in Chinese Esophageal Squamous Cell Carcinoma Patients With Definitive Chemoradiotherapy

**DOI:** 10.3389/fgene.2021.799663

**Published:** 2022-04-25

**Authors:** Honghai Dai, Yanjun Wei, Yunxia Liu, Jingwen Liu, Ruoying Yu, Junli Zhang, Jiaohui Pang, Yang Shao, Qiang Li, Zhe Yang

**Affiliations:** ^1^ Tumor Research and Therapy Center, Shandong Provincial Hospital Affiliated to Shandong First Medical University, Jinan, China; ^2^ Tumor Research and Therapy Center, Shandong Provincial Hospital Affiliated to Shandong University, Jinan, China; ^3^ Nanjing Geneseeq Technology Inc, Nanjing, China; ^4^ School of Public Health, Nanjing Medical University, Nanjing, China

**Keywords:** ESCC (Esophageal squamous cell carcinoma), *Keap1-Nrf2* pathway, *PI3K-Akt* pathway, chemoradiotherapy, pathway-based analysis

## Abstract

Esophageal squamous cell carcinoma (ESCC) is the major type of EC in China. Chemoradiotherapy is a standard definitive treatment for early-stage EC and significantly improves local control and overall survival for late-stage patients. However, chemoradiotherapy resistance, which limits therapeutic efficacy and treatment-induced toxicity, is still a leading problem for treatment break. To optimize the selection of ESCC patients for chemoradiotherapy, we retrospectively analyzed the clinical features and genome landscape of a Chinese ESCC cohort of 58 patients. *TP53* was the most frequent mutation gene, followed by *NOTCH1*. Frequently, copy number variants were found in *MCL1* (24/58, 41.4%), *FGF19* (23/58, 39.7%), *CCND1* (22/58, 37.9%), and *MYC* (20/58, 34.5%). *YAP1* and *SOX2* amplifications were mutually exclusive in this cohort. Using univariate and multivariate analyses, the *YAP1* variant and *BRIP1* mutant were identified as adverse factors for OS. Patients with *PI3K*-*Akt* pathway alterations displayed longer PFS and OS than patients with an intact *PI3K*-*Akt* pathway. On the contrary, two patients with *Keap1*-*Nrf2* pathway alterations displayed significantly shortened PFS and OS, which may be associated with dCRT resistance. Our data highlighted the prognostic value of aberrant cancer pathways in ESCC patients, which may provide guidance for better chemoradiotherapy management.

## Introduction

Esophageal carcinoma (EC) is the ninth most common cancer and remains the sixth leading cause of cancer death worldwide (Bray et al., 2018). Esophageal squamous cell carcinoma (ESCC) and Esophageal adenocarcinoma (EAC) are two major subtypes of EC and account for 90% of EC cases worldwide. On the other hand, different histological types of EC distributed varied around the world. ESCC contributes to 90% of all esophagus carcinomas each year in China, whereas EAC is mainly reported in North America and Europe (Abnet et al., 2018). Frequent consumption of hot beverages, a common lifestyle in China, results in a higher potential of ESCC, whereas people with gastroesophageal reflux, following a Western pattern diet, and with smoking behavior often have a higher risk of ADC (Dent et al., 2005). The 5-year survival rate of EC patients with only esophagus cancer is 47%, while the rate decreases to 25% if the tumor has spread to the surrounding organs or lymph nodes (Viale, 2020). Due to the poor prognosis and survival in EC, there is a strong demand for studying prognosis-related factors and seeking better treatment for patients with EC (Tustumi et al., 2016). The pathological pattern of Chinese EC provided us a unique opportunity to study the molecular mechanism underlying ESCC pathogenesis and disease outcomes.

Definitive chemoradiation therapy has been employed as the standard first-line therapy for ESCC patients. However, intolerance to radiotherapy and/or resistance to chemoradiotherapy was frequently observed with a high possibility of recurrence. The target therapy drug trastuzumab is the only HER2 monoclonal antibody approved by the FDA as a first-line drug along with chemotherapy for ESCC patients. Ramucirumab, an angiogenesis inhibitor that targets the VEGF/VEGFR2 pathway, has also been approved for EAC therapy (Yang et al., 2020). In addition, immunotherapy has been extensively evaluated in esophageal cancer. Nivolumab and pembrolizumab are two immune checkpoint inhibitors that target the PD-1/PD-L1 pathway approved by the FDA. Nivolumab (mOS = 10.9) has been confirmed to reduce the risk of death by 23% compared to chemotherapy alone (mOS = 8.4) in the phase 3 ATTRACTION-3 trial (mOS = 10.9) (Takahashi et al., 2021). These novel treatments have brought tremendous benefits to patients with a much longer survival time and better prognosis. Hence, the field of research on finding more targets for immune pharmaceuticals and targeted therapy is well worth exploring, and thus increasing the beneficial population.

It is well known that some signaling pathways altered across various tumor types, while others were highly associated with certain types of cancer, such as the oxidative stress response pathway in squamous cell carcinoma (Choe et al., 2021). For ESCC patients, definitive chemoradiotherapy is a standard therapy for non-resectable tumors. Pathways related to oxidative/electrophilic stress, like the cell cycle and *Keap1*-*Nrf*2 pathways, are therefore highly important for these patients to regulate exogenous stress from reactive oxygen species (ROS)/electrophiles induced by chemotherapy and radiotherapy. Here, we analyzed the alterations of ten canonical cancer-related pathways in this Chinese ESCC cohort (Sanchez-Vega et al., 2018). The ten pathways are cell cycle, *PI3 k*inase/Akt, *Keap1*-*Nrf2*, *Notch*, *p53*, *Myc*, *Hippo*, b-catenin/*Wnt*, RTK-RAS, and TGF*β* signaling. Some pathways significantly correlated with the prognosis, which might aid in stratifying patients for better treatment management.

## Materials and Methods

### Patients and Sample Collection

A total of 65 patients with ESCC were enrolled from the Tumour Research and Therapy Center, Shandong Provincial Hospital Affiliated to Shandong First Medical University, from 2016 to 2020 for retrospective analysis. Six patients were excluded from this study owing to their low-quality tissue samples, and one patient was excluded because no detectable mutation was found in this patient’s sample (Supplementary Figure S1). Eventually, 58 patients were included in the study. All patients were diagnosed with unresectable locally advanced ESCC or advanced ESCC (stages II-IV, American Joint Committee on Cancer, seventh edition) and underwent standard definitive chemoradiotherapy (dCRT). For each patient, a somatic formalin-fixed paraffin-embedded (FFPE) tissue biopsy was performed before definitive chemoradiotherapy. All tumor tissue samples with at least 10% tumor cell content were subjected to targeted panel sequencing using a 422-gene panel. This study was approved by the Ethical Review Board of the Shandong Provincial Hospital Affiliated to Shandong First Medical University.

### DNA Extraction and Library Preparation

The process from DNA extraction to library construction to target enrichment was performed in a CLIA-certified and CAP-accredited laboratory as previously described (Fang et al., 2019; Dai et al., 2020). In brief, genomic DNA from FFPE tissue was extracted using a QIAamp DNA FFPE Tissue Kit (Qiagen). DNA quantitation was then performed by using a QubitTM dsDNA HS Assay Kit for each sample, with its quality been identified by a NanoDropTM 2000 Spectrophotometer. Then we constructed the library for Illumina sequencing from fragmented dsDNA, using a KAPA HyperPrep kit (KAPA BIOSYSTEMS). The main steps of library preparation include end-repair and A-tailing, adapter ligation, and library amplification. The end-repair and A-tailing steps prepare end-repaired DNA, and 3’ A-tailing prepares double-stranded DNA. Adapter ligation attaches synthesized oligonucleotides as adapters to one or both ends of targeted DNA fragments. The final step of library preparation performs a low-bias and high-fidelity polymerase chain reaction (PCR) to amplify the targeted sequences carrying proper adapters, accompanied with an AMPure XP agent (Beckman Coulter) for purification. The customed xGen lockdown probes panel, containing 422 refined cancer-related genes, was further used to enrich the targeted genes. Subsequently, the prepared library was quantified using a KAPA Library Quantification Kit (KAPA BIOSYSTEMS), and the size distribution of each sample was calculated by Bioanalyzer 2100 (Agilent Technologies).

### DNA Sequencing With Quality Control

Targeted enriched libraries from the last step were sequenced using the Illumina HiSeq4000 Sequencing System to a mean coverage depth of at least 250×. The output BCL files (image data) from sequencing system were then demultiplexed and converted into readable FASTQ files by BCL2Fastq Conversion (version 1.8.4) from Illumina. Fastp (0.20.0; https://github.com/OpenGene/fastp/) was responsible for removing low-quality bases (base quality score Q30 < 30), trimming adapters, and read pruning. Qualified data were then mapped to the reference human genome (hg19 37d5) using a Burrows–Wheeler Aligner (BWA-mem, v0.7.12; https://github.com/lh3/bwa/) to produce bam files. The bam files were further sorted and then filtered into the final mapped file through the process of reads deduplication, local realignment, and base quality recalibration using Sambamba (v1.3; https://lomereiter.github.io/sambamba/) software. By comparing the consistency of SNP-associated signatures between tissue cell-free DNA and negative control in the Genome Analysis Toolkit (GATK 4.0.0; "https://software.broadinstitute.org/gatk/") contamination module, the samples were matched to each patient, as well as the DNA contamination score was estimate.

### Mutation Calling and Annotations

The fully qualified sequencing data were then processed to a series of software for single‐nucleotide variations (SNVs), insertion/deletion mutations, fusion, and copy number variation (CNV) detection. VarScan2 (Koboldt et al., 2012) was performed for detecting somatic mutations. Calls with a threshold of ≥1% mutant allele frequency (MAF) and ≥3 reads from both directions were retained. From these variant calls, SNPs in normal samples were filtered based on a list of sources, including dbSNP (Sherry et al., 2001), ClinVAR (Landrum et al., 2016), 1,000 Genome database (Genomes Project et al., 2015), 65,000 exomes project (ExAC) (Karczewski et al., 2017), COSMIC (v70) (Forbes et al., 2015), SIFT (Ng and Henikoff, 2003), and the laboratory’s SNP database of pre-existing population. ANNOVAR (Wang et al., 2010) was used to annotate all these SNVs. For somatic mutations, calls were removed if they were present in >1% populations in 1,000 Genome database or in ExAC. The resulting list was further filtered through an in-house mutation list of common sequencing errors. Additionally, a variant with >20% abundance in the normal sample, likely an artifact, was also removed from the mutation list. Structural variants were detected using FACTERA with default parameters (Newman et al., 2014). And the CNVs were detected by ADTEx (GPLv3; http://adtex.sourceforge.net/), both with default parameters. The threshold for CNV loss was 0.65 and 2.0 for the CNV gain.

### Mutation Description and Statistical Analysis

Oncoplots, constructed by R (4.0.3), were used to view the overall mutation landscape of ESCC patients in this study. Progression-free survival (PFS) was defined from the date of pathological diagnosis of esophageal carcinoma (EC) to the time of disease progression, worsening, or the last follow-up before progression. Overall survival (OS) started from EC diagnosis to the date of death or the last follow-up. The Kaplan–Meier method was used to estimate these two outcome measures among different genetic groups, different physiological populations, and selected pathways, followed by a stratified log-rank test for evaluating any differences. Subsequently, univariate Cox hazard models were further performed to define any prognostic factors affecting PFS and OS in this cohort. Statistically significant factors (*p*-value ≤0.1) defined in the single factor analysis were reviewed in detail. The beta coefficient in the pathway-related univariate analysis was the degree of change in the outcome (PFS or OS) for every 1-unit change in the number of pathway gene expression.

## Results

### Clinical Characteristics and Mutation Landscape of ESCC Patients

The basic characteristic of 58 enrolled ESCC patients is shown in Supplementary Table S1. More than half of patients in the cohort were older than 60 years (55.17%), with a median age of 63 (range: 41–83) years. Forty-six patients (79.31%) were male, and only 12 (20.69%) were female. Around sixty-eight percent (39/58) of the patients were smokers, and fifty percentage had a history of alcohol consumption (29/58). More than half of the patients were diagnosed with stage III (36/58, 62.07%) ESCC, and 16 patients (16/58, 27.59%) were in stage II, with additional six patients (6/58, 10.34%) in stage IV.

**FIGURE 1:**
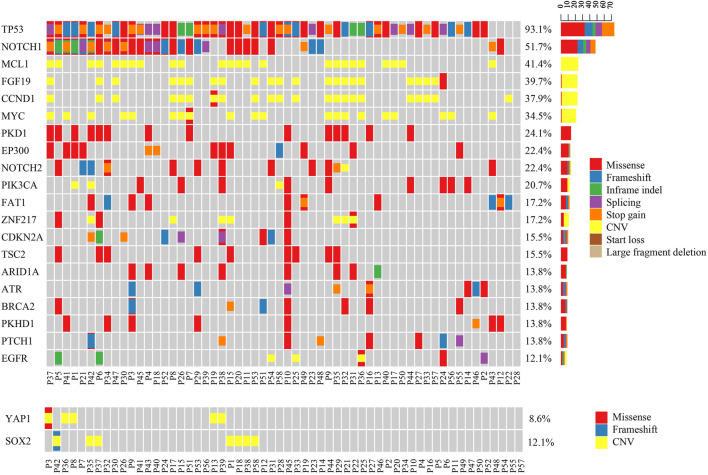
Mutational pattern in Chinese ESCC patients. The upper oncoplot shows the mutational landscape of patients in this cohort. The lower oncoplot shows that *YAP1* gain and *SOX2* gain are mutually exclusive to each other. No patient had both amplifications at the same time.

In these Asian ESCC patients, *TP53* (54/58, 93.1%) was the most frequent mutation gene, followed by *NOTCH1* (30/58, 51.7%) (Figure 1). Amplification of *MCL1* (24/58, 41.4%), *FGF19* (23/58, 39.7%), *CCND1* (22/58, 37.9%), and *MYC* (20/58, 34.5%) was the four dominant types of CNV identified in this ESCC cohort. As previously mentioned, *FGF19* and *CCND1* were often co-amplified since they were both at adjacent locations on chromosome 11q13. Interestingly, *YAP1* and *SOX2* amplifications were mutually exclusive to each other in these ESCC patients (Figure 1). A similar negative correlation of the protein expression level in *YAP1* and *SOX2* was also found *in vivo* and *in vitro* of pancreatic neoplastic cells (Seo et al., 2013; Murakami et al., 2019).

### Gene Alterations Associated With Disease Outcomes in ESCC Patients

**FIGURE 2:**
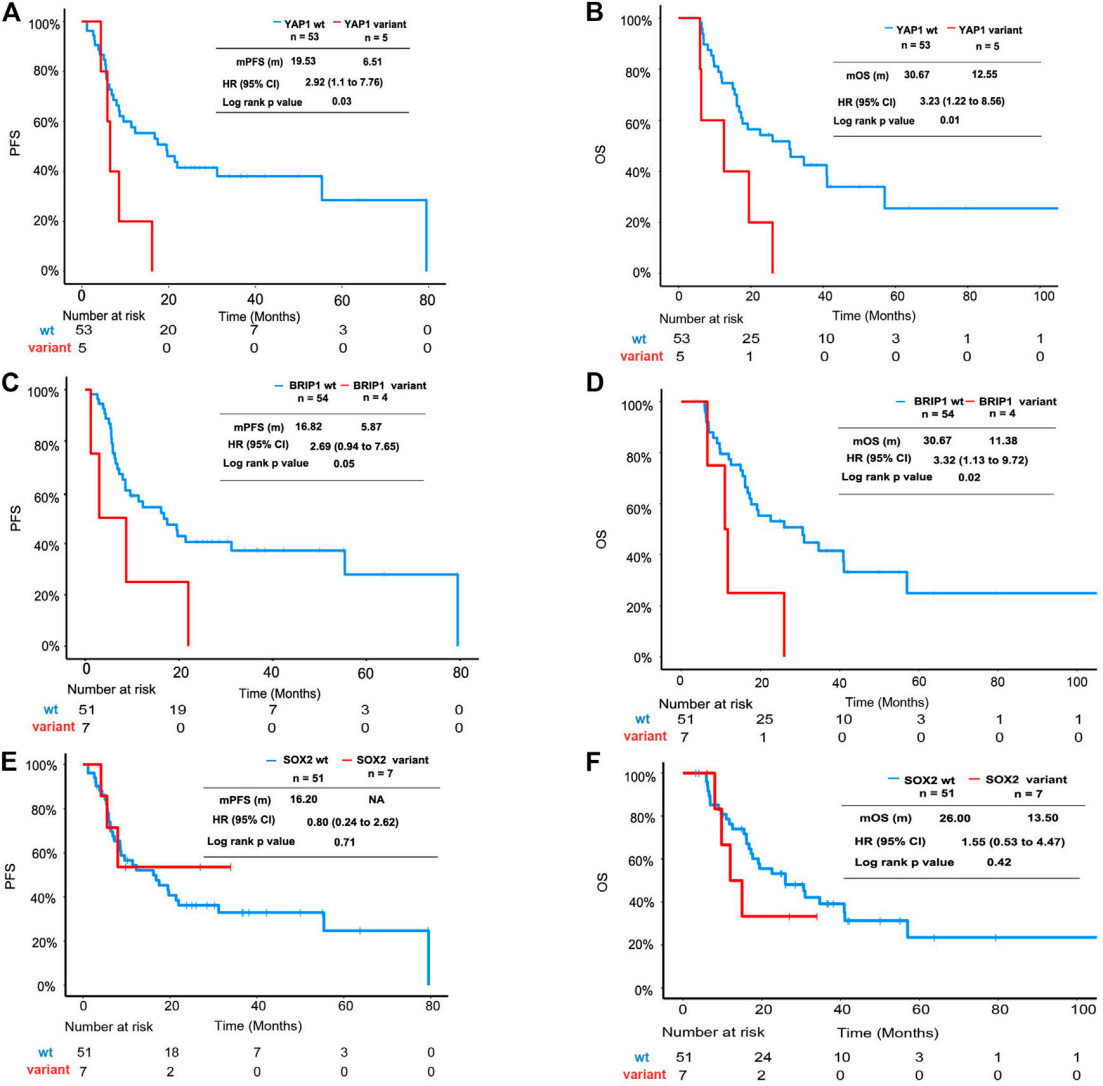
Survival analysis of ESCC patients with *YAP1* mutation, *BRIP1* variation, and *SOX2* mutation. **(A)** Kaplan–Meier plot showing PFS of the subgroup patients with *YAP1* mutation versus patients without *YAP1* mutation. **(B)** Kaplan–Meier plot showing OS of the subgroup patients with *YAP1* mutation versus patients without *YAP1* mutation. **(C)** Kaplan–Meier plot showing PFS of the subgroup patients with *BRIP1* mutation versus patients without *BRIP1* variation. **(D)** Kaplan–Meier plot showing OS of the subgroup patients with *BRIP1* mutation versus patients without *BRIP1* variation. **(E)** Kaplan–Meier plot showing PFS of the subgroup patients with *SOX2* amplification versus patients without *SOX2* mutation. **(F)** Kaplan–Meier plot showing OS of the subgroup patients with *SOX2* amplification versus patients without *SOX2* mutation.

In this cohort, the *YAP1* variant and *BRIP1* mutant were identified as adverse factors for PFS and OS in univariate analysis. In multivariate analysis, the *YAP1* variant and *BRIP1* mutant were significantly associated with OS but not with PFS (Supplementary Table S2). The Kaplan–Meier plot revealed that median PFS (mPFS) and median OS (mOS) of patients with the *YAP1* variant was 8.61 and 12.55 months, respectively, which were significantly shorter than that of *YAP1* wild-type patients (Figures 2A,B). ESCC patients with the *BRIP1* mutant also displayed worse outcomes than ones with the *BRIP1* wild type, achieving an mPFS of 5.87 months and an mOS of 11.38 months (Figures 2C,D). *SOX2* amplification, which was mutually exclusive to *YAP1* in this cohort, did not reach statistical significance in univariate analysis (Figures 2E,F).

### Prognosis Value of Cancer-Associated Pathways in ESCC

**FIGURE 3:**
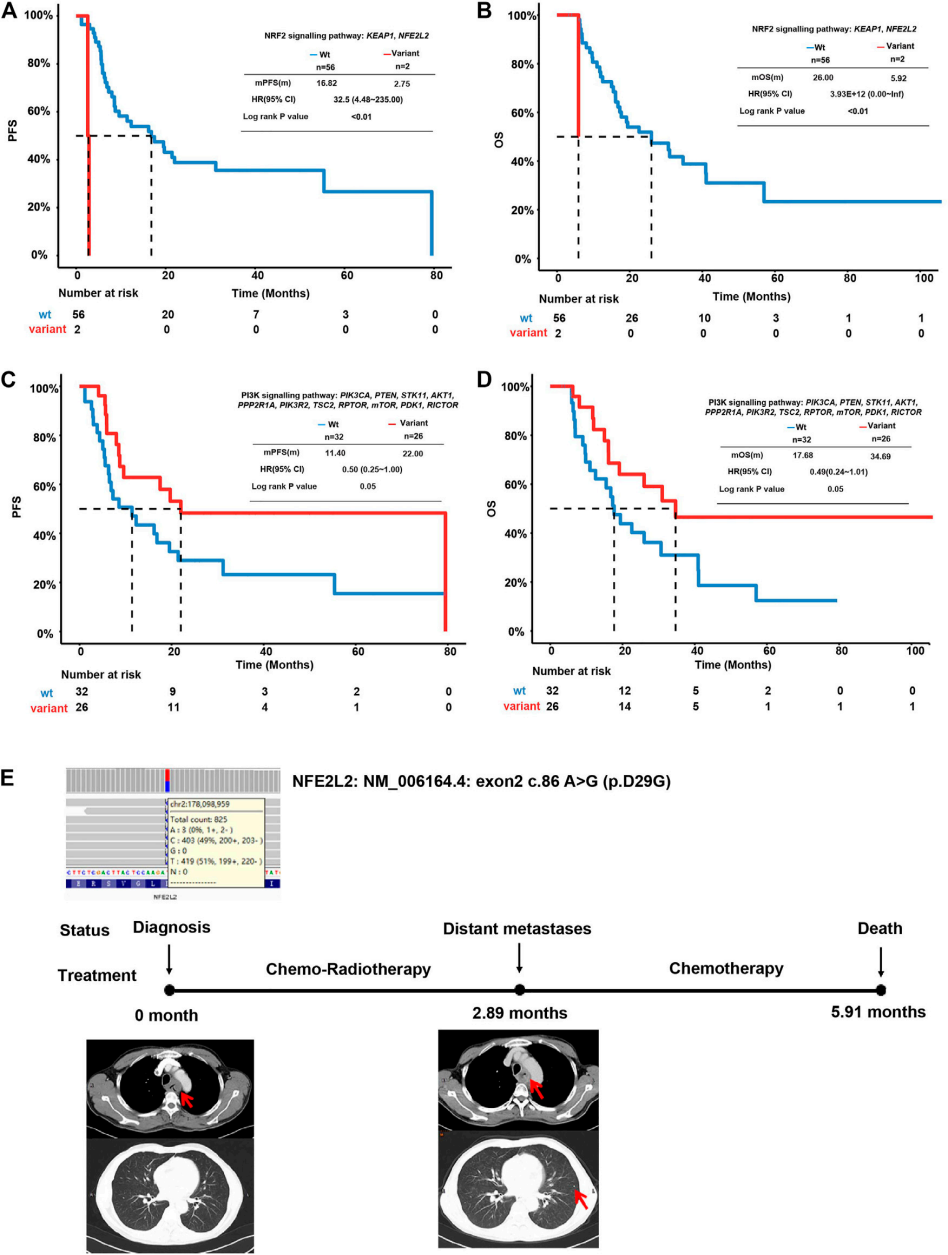
Survival analysis of ESCC patients with altered oncogenic pathways. **(A)** Kaplan–Meier plot for PFS of ESCC patients with an intact or altered *Keap1-Nrf2* pathway. **(B)** Kaplan–Meier plot for OS of ESCC patients with an intact or altered Keap1-Nrf2 pathway. **(C)** Kaplan–Meier plot for PFS of ESCC patients with an intact or altered PI3K-Akt pathway. **(D)** Kaplan–Meier plot for OS of ESCC patients with an intact or altered PI3K-Akt pathway. **(E)** Representative case of a patient with *Keap1-Nrf2* pathway alterations.

Pathway analysis was performed according to the genes in ten cancer-associated pathways in the literature (Supplementary Table S3) (Sanchez-Vega et al., 2018). The individual genes in each included pathway are listed in Supplementary Table S1. Around 93% of this EC cohort harbored TP53 signaling pathway alterations. Altered NOTCH (72.41%), RTK-RAS (68.97%), and cell cycle (53.45%) pathway genes were identified in more than 50% of the total cases (Supplementary Figure S2 and Supplementary Table S3). EC patients with mutations in the *Keap1*-*Nrf2* pathway had much shorter (*n* = 2, mPFS = 2.75, beta = 3.48, *p* < 0.0001, HR (95% CI = 32.5 (4.48–235)) PFS than wild-type patients (*n* = 56, mPFS = 16.2) (Figure 3A). Similarly, mutations in this pathway also increased the risk of unfavorable OS (*n* = 2, beta coefficient = 29, *p* < 0.0001) compared to the wild-type counterpart (*n* = 56, mOS = 26.0) (Figure 3B). This observation was also validated using an independent cohort of 88 ESCC patients with OS information (Song et al., 2014). As shown in Supplementary Figure S3, seven patients had the altered *Keap1*-*Nrf2* pathway with a significantly shortened OS compared to patients with the intact *Keap1*-*Nrf2* pathway (*p* = 0.039).

In contrast to *Keap1*-*Nrf2* pathway aberrations, patients with mutations in the *PI3K*-*Akt* pathway displayed a longer PFS (*n* = 26, mPFS = 22, beta = 0.74, *p* = 0.0337, and HR (95% CI) = 0.48 (0.24–0.96) and longer OS (*n* = 26, mOS = 34.69, beta = 0.71, *p* = 0.0495, and HR (95% CI) = 32.5 (0.24–1.01). Comparatively, wild-type patients achieved a shorter PFS (*n* = 32, mPFS = 9.8) and OS (*n* = 32, mOS = 17.68) (Figures 3C,D). In patients with *PI3K-Akt* pathway alterations, three were found with altered *PTEN* and seven were found with altered *PIK3CA*. Patients with *PIK3CA* mutation tend to have longer PFS and OS than patients with wild-type *PIK3CA.* The altered *PTEN* did not show association with PFS or OS in this cohort (Supplementary Figure S4).

A representative case of an ESCC patient with *NFE2L2* mutation is shown in Figure 3E. The patient was a 49-year-old male diagnosed with stage IV ESCC. He was identified with *NFE2L2* D29G mutation at an allele frequency (AF) of 48.19% before treatment. The *RB1* frameshift mutation and *TP53* G262V were identified at an AF of 52.13 and 37.25%, respectively, at the same time. The tumor quickly progressed after 2.89 months of dCRT and metastasized to distant lymph nodes. Eventually, the patient died after 5.91 months of chemoradiotherapy and chemotherapy.

## Discussion

In this study, we retrospectively studied the clinical features and cancer genomes of 58 patients with inoperable ESCC tumors, intending to identify prognostic biomarkers for Chinese ESCC patients. Among all the baseline clinical characteristics, gender appeared to be an independent prognostic factor, which was in accord with the previous study (Pandeya et al., 2013). The high frequency of gene amplification was another genetic feature observed in esophageal squamous cell carcinoma. In our cohort, 75.9% (44/58) patients had at least one gene amplified. *MCL1* (24/58, 41.4%), *FGF19* (22/58, 37.9%), *CCND1* (22/58, 37.9%), and *MYC* (20/58, 34.5%) were the four dominant amplified genes. Besides, *YAP1* and *SOX2* were found to be exclusively amplified in different patients in this cohort. By further reviewing the prognosis of patients with/without *YAP1* and *SOX2* amplification, patients without double amplification were found to have the best PFS and OS. The group of patients with *SOX2* amplification and the group with *YAP1* amplification both obtained shorter PFS and OS, which consistent with the previous study (Dai et al., 2020).

Interestingly, the exclusion of *YAP1* amplification and *SOX2* amplification was only reported in one mouse model study that Yap loss intended to induce acute metabolic stress, leading to epigenetic reprogramming with *SOX2* upregulation (Murakami et al., 2019). Most other studies showed that *YAP1* is co-amplified with *SOX2* by *YAP1* binding to *SOX2*’s enhancer region, and *SOX2* may in turn restore *YAP1* by antagonizing the Hippo pathway in maintaining cell stemness and leading to poor prognosis. The cooperation of *YAP1* and *SOX2* was detected in various cancer types, including osteosarcoma, urothelial cancer, and HNSCC (head and neck squamous cell carcinoma) (Murakami et al., 2019; Omori et al., 2019). Thus, behind the scenes of mutual exclusion for *SOX2* amplification and *YAP1* amplification of these patients in this study, there lies a unique unknown molecular mechanism of ESCC tumorigenesis, distinguished from other cancer types, which needs further investigation.

Of the two pathways identified as potential prognostic biomarkers of ESCC, the *Keap1*-*Nrf2* pathway is known for inducing chemoradioresistance (Taguchi and Yamamoto, 2017; Zhang et al., 2018). One of the major roles of *Nrf2* is to initiate cytoprotective responses under oxidant stress by binding to and activating the antioxidant response element (ARE) in the modular regions of its downstream targets (Kansanen et al., 2013). In addition, *Nrf2* promotes cell proliferation and metabolic reformation by triggering metabolic genes. On the other hand, *Keap1* can inhibit the *Keap1*-*Nrf2* pathway by suppressing the expression of *Nrf2*. Under oxidative stress and electrophilic stress, the confirmation of *Keap1* is reconstructed due to alterations in its cysteine residues. Newly synthesized *Nrf2* can bypass *Keap1* and translocate into the nucleus by *Keap1* protein inactivation or *Keap1*-*Nrf2* complex disruption (Kansanen et al., 2013). Here, the two patients carrying mutations in the *Keap1*-*Nrf2* pathway exhibit poor disease outcomes with shorter PFS and OS compared to *Keap1*-*Nrf2* pathway wild-type patients. The rapid progression of patients carrying abnormalities in the *Keap1*-*Nrf2* pathway in other cancer types was reported in several studies (Zoja et al., 2014; Goeman et al., 2019). Due to the limited number of patients with altered *Keap1*-*Nrf2* pathways in this study, further research is needed to identify whether activating mutations of the *Keap1*-*Nrf2* pathway is a potential chemo-radioresistance–related biomarker for patients receiving dCRT therapy.

*PIK3CA* mutation was a commonly reported factor for treatment and prognosis in ESCC patients, but conflicting conclusions were drawn across studies (Wada et al., 2006; Shigaki et al., 2013; Wang et al., 2014). Our studies showed a favorable prognosis among the patients with muted *PI3K* pathways. The PI3K-AKT pathway is considered one of the master regulators for cancer and ideal targets for anticancer drugs (Yang et al., 2019). It is known to play an important role in the development and progression of many solid cancers (Song et al., 2011; Jiao and Nan, 2012; Vredeveld et al., 2012). Further *in vivo* study or expansion of cohort size was needed to confirm our results.

## Data Availability

The data has been uploaded to the GSA database. The accession ID is HRA002194. https://ngdc.cncb.ac.cn/gsa-human/s/4I2LAq98.
