# Supplementary figures and images for: Pathway-Based Analysis Revealed the Role of Keap1-Nrf2 Pathway and PI3K-Akt Pathway in Chinese Esophageal Squamous Cell Carcinoma Patients With Definitive Chemoradiotherapy
Source: Front Genet. 2022 Apr 25;12:799663. doi: 10.3389/fgene.2021.799663 (PMC9081370; doi:10.3389/fgene.2021.799663)

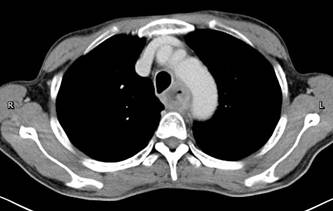

Supplement: Supplementary file 1 [file Image3.JPEG]

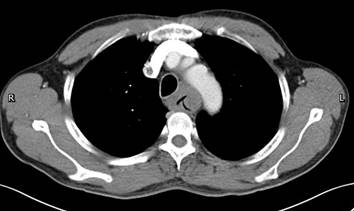

Supplement: Supplementary file 3 [file Image1.JPEG]

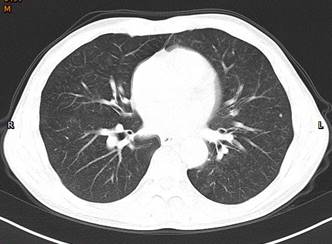

Supplement: Supplementary file 4 [file Image4.JPEG]

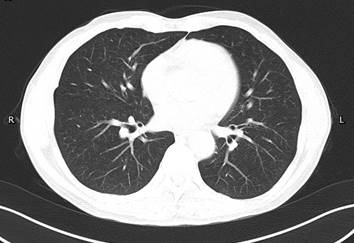

Supplement: Supplementary file 5 [file Image2.JPEG]
