# Supplementary material for: Pathway-Based Analysis Revealed the Role of Keap1-Nrf2 Pathway and PI3K-Akt Pathway in Chinese Esophageal Squamous Cell Carcinoma Patients With Definitive Chemoradiotherapy
Source: Front Genet. 2022 Apr 25;12:799663. doi: 10.3389/fgene.2021.799663 (PMC9081370; doi:10.3389/fgene.2021.799663)

Table S1. List of ten pathway-associated genes.

| **Cancer-associated pathways** | **Associated genes** |
| --- | --- |
| **Cell cycle pathway** | CCND1, RB1, CDKN2A, CDKN1B, CDK4, CDK6, CCNE1 |
| **Hippo pathway** | FAT1, YAP1, NF2 |
| **MYC pathway** | MYC, MYCN, MAX |
| **NOTCH pathway** | NOTCH2, KDM5A, NOTCH1, EP300, CREBBP, FBXW7, HDAC2 |
| **NRF2 pathway** | NFE2L2, KEAP1 |
| **PI3K pathway** | PTEN, PIK3CA, STK11, AKT1, PPP2R1A, PIK3R2, TSC2, RPTOR, MTOR, PDK1, RICTOR |
| **TGFB pathway** | TGFBR2, SMAD4 |
| **RTK RAS pathway** | KIT, ERBB4, ALK, ROS1, MET, PTPN11, BRAF, PDGFRA, FGFR2, FGFR4, ARAF, RET, IGF1R, ERBB2, FLT3, PDGFRB, EGFR, ERBB3, MAP2K2, NF1, KRAS, FGFR3, CBL, NTRK1, NRAS, FGFR1 |
| **TP53 pathway** | TP53, ATM, CHEK2, MDM4, MDM2 |
| **WNT pathway** | LZTR1, APC, CHD4, CTNNB1, AXIN2, AMER1, CHD8 |

Figure S1. Study flowchart


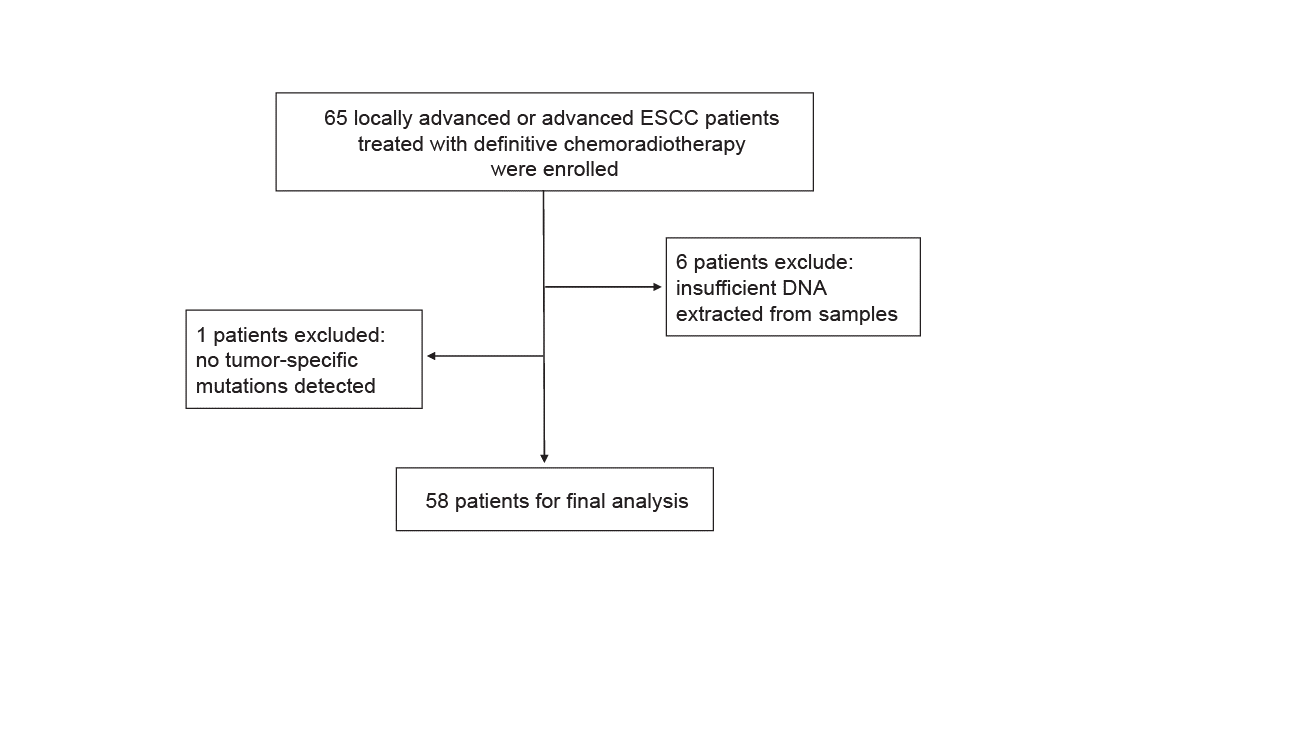


Figure S2. The distribution of mutated genes among ten tumor-related pathways of ESCC patients. Each column represented one patient. The types of mutations were indicated by the color shown on the right.


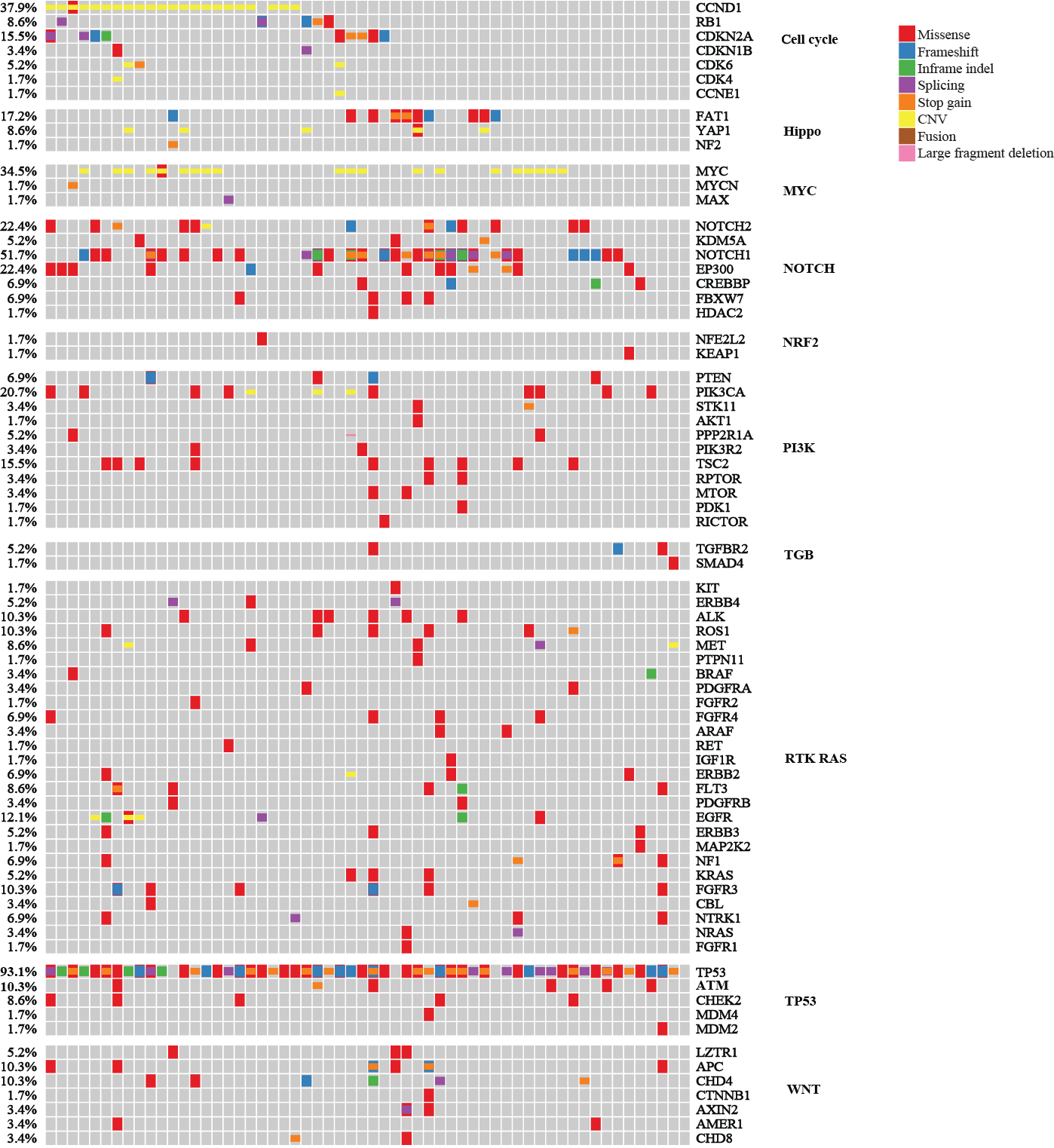


Figure S3. Validation of *NRF2* pathway using an independent ESCC cohort with 88 patients. Kaplan-Meier plot showed OS of the subgroup patients with altered *NRF2* pathway versus patients without.


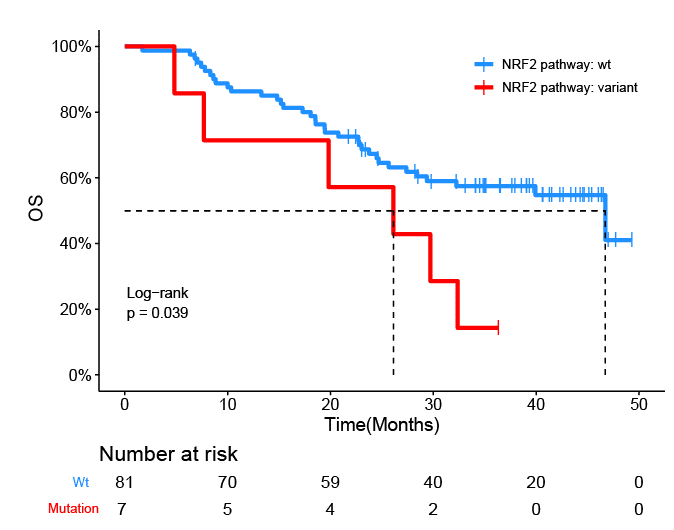


Figure S4. Survival analysis of EC patients with *PTEN* and *PIK3CA* alterations. A. Kaplan-Meier plot showed PFS and OS of the subgroup patients with *PTEN* mutation versus patients without *PTEN* mutation. B. Kaplan-Meier plot showed PFS and OS of the subgroup patients with *PIK3CA* mutation versus patients without *PIK3CA* mutation


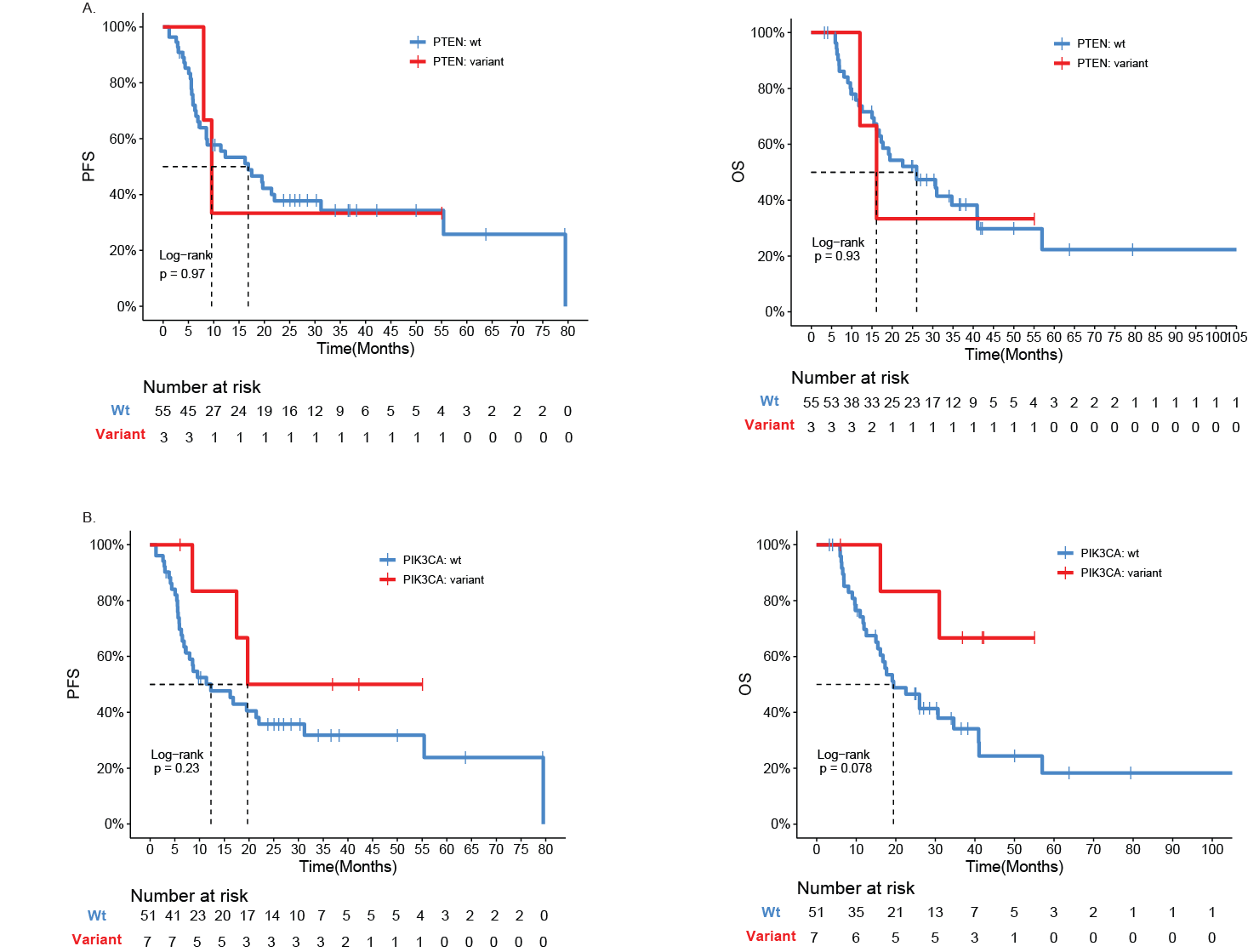

Supplement: Supplementary file 2 [file Table1.DOCX]
