# Supplementary material for: Pathway-Based Analysis Revealed the Role of Keap1-Nrf2 Pathway and PI3K-Akt Pathway in Chinese Esophageal Squamous Cell Carcinoma Patients With Definitive Chemoradiotherapy
Source: Front Genet. 2022 Apr 25;12:799663. doi: 10.3389/fgene.2021.799663 (PMC9081370; doi:10.3389/fgene.2021.799663)
Supplement: Supplementary file 6 [file Table2.DOCX]

| Table 1 The basic characteristics of ESCC patients | | | |  | | | |  |  |  |
| --- | --- | --- | --- | --- | --- | --- | --- | --- | --- | --- |
| Characteristics | | | | No. of Patients (%) | | | |  |  |  |
| Age (years) | | | | | | | |  |  |  |
| ≤60 | | | | 26 (44.83%) | | | |  |  |  |
| >60 | | | | 32 (55.17%) | | | |  |  |  |
| Median (range) | | | | 63 (41~83) | | | |  |  |  |
| Gender | | | | | | | |  |  |  |
| Male | | | | 46 (79.31%) | | | |  |  |  |
| Female | | | | 12 (20.69%) | | | |  |  |  |
| Smoking history | | | | | | | |  |  |  |
| Never | | | | 19 (32.76%) | | | |  |  |  |
| Ever | | | | 39 (67.24%) | | | |  |  |  |
| Alcohol consumption | | | | | | | |  |  |  |
| Never | | | | 29 (50.00%) | | | |  |  |  |
| Ever | | | | 29 (50.00%) | | | |  |  |  |
| Stage | | | | | | | |  |  |  |
| Ⅱ | | | | 16 (27.59%) | | | |  |  |  |
| Ⅲ | | | | 36 (62.07%) | | | |  |  |  |
| Ⅳ | | | | 6 (10.34%) | | | |  |  |  |
| T stage | | | | | | | |  |  |  |
| pT1 | | | | 2 (3.44%) | | | |  |  |  |
| pT2 | | | | 5 (8.62%) | | | |  |  |  |
| pT3 | | | | 41 (70.69%) | | | |  |  |  |
| pT4 | | | | 10 (17.24%) | | | |  |  |  |
| N stage | | | | | | | |  |  |  |
| pN0 | | | | 18 (31.03%) | | | |  |  |  |
| pN1-N3 | | | | 40 (68.97%) | | | |  |  |  |
| Table 2. Univariate and multivariate analysis of genes effecting PFS and OS in ESCC patient. -: not significant in univariate analysis therefore not included in the multivariate analysis. | | | | | | | | | | |
| Factors | PFS | | | | | OS | | | | |
|  | Univariate analysis | | Multivariate analysis | | | Univariate analysis | | | Multivariate analysis | |
|  | HR (95% CI) | P value | HR (95% CI) | | P value | HR (95% CI) | P value | | HR (95% CI) | P value |
| Stage | 1.92 (1.05~3.52) | 0.035 | 1.31 (0.70~2.43) | | 0.398 | 1.51  (0.82~2.78) | 0.185 | | - | - |
| YAP1  (variant vs wt) | 2.92 (1.1~7.76) | 0.025 | 2.50 (0.88~7.04) | | 0.084 | 2.30  (1.22~8.56) | 0.013 | | 3.06  (1.08~8.66) | 0.035 |
| BRIP1  (mutant vs wt) | 2.69 (0.94~7.65) | 0.054 | - | | - | 3.32  (1.13~9.72) | 0.02 | | 5.16  (1.65~16.10) | 0.005 |
| SOX2  (variant vs wt) | 0.80 (0.24~2.62) | 0.71 | - | | - | 1.55  (0.53~4.47) | 0.42 | |  |  |

| Table 3. Potential prognostic value of the ten tumor related pathways in ESCC patients treated with dCRT. | | | | | | | | |
| --- | --- | --- | --- | --- | --- | --- | --- | --- |
| Signaling pathway | PFS | | OS | | Numbers of patients | Proportion |  |  |
|  | HR (95% CI) | p value | HR (95% CI) | p value |  |  |  |  |
| NRF2 | 32.5 (4.48~235) | <0.001 | 3.93e+12 (0~Inf) | <0.001 | 2 | 3.45% |  |  |
| PI3K | 0.50 (0.25~1.00) | 0.047 | 0.49 (0.24~1.01) | 0.049 | 26 | 44.83% |  |  |
| Cell cycle | 0.93 (0.48~1.80) | 0.82 | 0.90 (0.45~1.78) | 0.75 | 31 | 53.45% |  |  |
| Hippo | 1.62 (0.78~3.40) | 0.2 | 1.57 (0.742~3.32) | 0.23 | 13 | 22.41% |  |  |
| MYC | 1.69 (0.87~3.29) | 0.12 | 1.73 (0.87~3.45) | 0.11 | 22 | 37.93% |  |  |
| NOTCH | 0.65 (0.32~1.31) | 0.23 | 0.76 (0.36~1.6) | 0.47 | 42 | 72.41% |  |  |
| TGFβ | 0.55 (0.13~2.30) | 0.4 | 0.63 (0.15~2.65) | 0.53 | 4 | 6.90% |  |  |
| RTK RAS | 0.78 (0.39~1.56) | 0.48 | 0.81 (0.40~1.64) | 0.56 | 40 | 68.97% |  |  |
| TP53 | 0.47 (0.14~1.55) | 0.21 | 0.78 (0.24~2.58) | 0.69 | 54 | 93.10% |  |  |
| WNT | 0.91 (0.43~1.90) | 0.79 | 0.83 (0.38~1.79) | 0.63 | 15 | 25.86% |  |  |
