# Supplementary material for: Pathway-Based Analysis Revealed the Role of Keap1-Nrf2 Pathway and PI3K-Akt Pathway in Chinese Esophageal Squamous Cell Carcinoma Patients With Definitive Chemoradiotherapy
Source: Front Genet. 2022 Apr 25;12:799663. doi: 10.3389/fgene.2021.799663 (PMC9081370; doi:10.3389/fgene.2021.799663)
Supplement: Supplementary file 7 [file Table3.DOCX]

**Pathway-based analysis revealed the role of Keap1-Nrf2 pathway and PI3K-Akt pathway in Chinese Esophageal Squamous Cell Carcinoma patients with definitive chemoradiotherapy**

Honghai Dai^1^, Yanjun Wei^2^, Yunxia Liu^1^, Jingwen Liu^3^, Ruoying Yu^3^, Junli Zhang^3^, Jiaohui Pang^3^, Yang Shao^3,4^, Qiang Li1^＊^, Zhe Yang1^＊^

1. Tumor Research and Therapy Center, Shandong Provincial Hospital Affiliated to Shandong First Medical University, Jinan, Shandong, 250021, China
2. Tumor Research and Therapy Center, Shandong Provincial Hospital Affiliated to Shandong University, Jinan, China
3. Nanjing Geneseeq Technology Inc. Nanjing, Jiangsu, 210032, China
4. School of Public Health, Nanjing Medical University, Nanjing, Jiangsu, 210029, China

*Correspondence:

Qiang Li, Tumor Research and Therapy Center, Shandong Provincial Hospital Affiliated to Shandong First Medical University, Jinan, Shandong, 250021, China. Email: lq1211@126.com

Zhe Yang, Tumor Research and Therapy Center, Shandong Provincial Hospital Affiliated to Shandong First Medical University, Jinan, Shandong, 250021, China. Email: [sdslyyyz@sina.com](mailto:sdslyyyz@sina.com)

**Abstract**

Esophageal squamous cell carcinoma(ESCC) is the major type of EC in China. Chemoradiotherapy is a standard definitive treatment for early-stage EC and significantly improves local control and overall survival for late-stage patients. However, chemoradiotherapy resistance, which limited therapeutic efficacy and treatment-induced toxicity is still a leading problem for treatment break. To optimize the selection of ESCC patients for chemoradiotherapy, we retrospectively zanalyzed the clinical features and genome landscape of a 58 Chinese ESCC patients’ cohort. *TP53* was the most frequent mutation gene, followed by *NOTCH1*. Frequently copy number variants were found in *MCL1* (24/58, 41.4%), *FGF19* (23/58, 39.7%), *CCND1* (22/58, 37.9%) and *MYC* (20/58, 34.5%). *YAP1* amplification and *SOX2* amplifications were mutually exclusive in this cohort. Using univariate and multivariate analysis, *YAP1* variant and *BRIP1* mutant were identified as adverse factors for OS. Patients with *PI3K*-*Akt* pathway alterations displayed longer PFS and OS than patients with intact *PI3K*-*Akt* pathway. On the contrary, two patients with *Keap1*-*Nrf2* pathway alterations displayed significantly shorten PFS and OS, which may associate with dCRT resistance. Our data highlighted the prognostic value of aberrant cancer pathways in ESCC patients, which may provide guidance for better chemoradiotherapy management.

**Introduction**

Esophageal carcinoma(EC) is the ninth most common cancer and remains the sixth leading cause of cancer death worldwide[1]. Esophageal Squamous Cell Carcinoma (ESCC) and Esophageal Adenocarcinoma (EAC) are two major subtypes of EC and account for 90% EC cases worldwide. On the other hand, different histological types of EC distributed varied around the world. ESCC occupied 90% of all esophagus carcinoma each year in China, whereas EAC mainly rise in North America and Europe[2]. Frequently consumption of hot beverages, a common lifestyle in China, results in a higher potential of ESCC. Whereas people with gastro-oesophageal reflux, western pattern diet, and smoking often lead to a higher risk of ADC[3]. The five-year survival rate of EC patients with only esophagus-located cancer is 47% while the rate decrease to 25% if the tumor has spread to surrounding organs or lymph nodes[4] Due to the poor prognosis and survival in EC, there is a strong demand for studying prognosis-related factors and seeking better treatment for patients with EC[5]. The pathological pattern of Chinese EC provided us a unique opportunity to study the molecular mechanism underlying ESCC pathogenesis and disease outcomes.

Definitive chemoradiation therapy has been employed as the standard first-line therapy for ESCC patients. However, intolerance to radiotherapy and/or resistance to chemoradiotherapy were frequently observed with a high possibility of recurrence. Target therapy drugs trastuzumab is the only HER2 monoclonal antibody approved by FDA as a first-line drug along with chemotherapy for ESCC patients. Ramucirumab, which is an angiogenesis inhibitor that targets VEFG/VEFGR2 pathway, has also been approved for EAC patient[6]. In addition, immunotherapy has been extensively evaluated in esophageal cancer. Nivolumab and Pembrolizumab are two immune checkpoint inhibitors that target PD-1/PD-L1 pathway approved by FDA. Nivolumab (mOS = 10.9) has been confirmed to reduce the risk of death by 23% compared to chemotherapy alone (mOS = 8.4) in the phase 3 ATTRACTION-3 trial (mOS = 10.9)[7]. These novel treatments have brought tremendous benefits to patients with a much longer survival time and better prognosis. Hence the field of research on finding more targets for immune pharmaceuticals and targeted therapy is well worth exploring, increasing beneficial population.

It is well known that some signaling pathways altered across various tumor types, while others were highly associated with certain types of cancer, such as the oxidative stress response pathway in squamous cell carcinoma[8]. For ESCC patients, definitive chemoradiotherapy is a standard therapy for nonresectable tumors. Pathways related to oxidative/electrophilic stress, like cell cycle and *Keap1*-*Nrf*2 pathway, are therefore highly important for these patients to regulate exogenous stress from reactive oxygen species (ROS)/ electrophile induced by chemotherapy and radiotherapy. Here, we analyzed the alterations of ten canonical cancer-related pathways in this Chinese ESCC cohort[9]. The ten pathways are cell cycle, *PI3K*inase/Akt, *Keap1*-*Nrf2*, *Notch*, *p53*, *Myc*, *Hippo*, b-catenin/*Wnt*, RTK-RAS, and TGFb signaling. Some pathways significantly correlated with the prognosis, which might aid in stratifying patients for better treatment management.

**Method**

**Patients and sample collection**

A total of 65 patients with ESCC were enrolled from Tumour Research and Therapy Center, Shandong Provincial Hospital Affiliated to Shandong First Medical University from 2016 to 2020 for retrospective analysis. Six patients were excluded from this study owing to their low-quality tissue samples, and 1 patient was excluded because no detectable mutation was found in this patient’s sample(Figure S1). Eventually, 58 patients were included in the study. All patients were diagnosed with unresectable locally advanced ESCC or advanced ESCC (Stage II-IV, American Joint Committee on Cancer, 7th edition) and underwent standard definitive chemoradiotherapy(dCRT) treatment. For each patient, a somatic formalin-fixed paraffin-embedded (FFPE) tissue biopsy was collected before definitive Chemoradiotherapy. All tumor tissue samples with at least 10% tumor cell content were subjected for targeted panel sequencing using a 422-gene panel. This study was approved by the Ethical Review Broad of the Shandong Provincial Hospital Affiliated to Shandong First Medical University.

**DNA extraction and library preparation**

The process from DNA extraction, library construction to target enrichments was performed in a CLIA certified and CAP-accredited laboratory as previously described[10; 11]. In brief, genomic DNA from FFPE was extracted using QIAamp DNA FFPE Tissue Kit (Qiagen). DNA quantitation was then tested by QubitTM dsDNA HS Assay Kit for each sample, with its quality been identified by NanoDropTM 2000 Spectrophotometers. Then we constructed the library for Illumina sequencing from fragmented dsDNA, using the KAPA HyperPrep kits (KAPA BIOSYSTEMS). The main steps of library preparation include end repair and A-tailing, adaptor ligation, and library amplification. The end-repair and A-tailing step prepare end-repaired and 3’ A-tailing double-strand DNA. Adaptor ligation attaches synthesis oligonucleotides as adaptors to one or both ends of targeted DNA fragments. The final step of library preparation performs a low-bias and high-fidelity polymerase chain reaction (PCR) to amplify the targeted sequences carrying proper adapters, accompanied with AMPure XP agent (Beckman Coulter) for purification. Customed xGen lockdown probes panel, containing 422 refined cancer-related genes were further used to enrich the targeted genes. Subsequently, we quantified the prepared library with KAPA Library Quantification Kit (KAPA BIOSYSTEMS), and calculated the size distribution of each sample by Bioanalyzer 2100(Agilent Technologies).

**DNA Sequencing with Quality Control**

Targeted enriched libraries from the last step were sequenced on the Illumina HiSeq4000 Sequencing System to a mean coverage depth of at least 250×. The output BCL files (image data) from sequencing were then demultiplexed and converted into readable FASTQ files by BCL2Fastq Conversion (Version 1.8.4) from Illumina. Fastp(0.20.0; <https://github.com/OpenGene/fastp>) was responsible for removing low-quality bases(base quality score Q30 < 30), trimming adaptors and read pruning. Qualified data were then mapped to reference human genome (hg19 37d5) using Burrows-Wheeler Aligner (BWA-mem, v0.7.12; <https://github.com/lh3/bwa>) to produce bam files. The bam files were further sorted and then filtered into the final mapped file through the process of reads de duplication, local realignment, and base quality recalibration using the Sambamba(v1.3; <https://lomereiter.github.io/sambamba/>) software. By comparing the consistency of SNP-associated signatures between tissue cell-free DNA and negative control in the Genome Analysis Toolkit (GATK 4.0.0; <https://software.broadinstitute.org/gatk/>) Contamination module, we were able to match samples to each patient, as well as to estimate the DNA contamination score.

**Mutation calling and Annotations**

The fully qualified sequencing data were then processed to a series of software for single‐nucleotide variations (SNVs), insertion/deletion mutations, fusion, and copy number variation (CNV) detection. VarScan2[12]was performed for detecting somatic mutation. Calls with a threshold of ≥1% mutant allele frequency (MAF), meanwhile ≥ 3 reads from both directions were retained. From these variant calls, SNPs in normal samples were filtered based on a list of sources, including dbSNP[13], ClinVAR[14], 1,000 Genome Database[15], 65000 exomes project (ExAC)[16], COSMIC (v70)[17], SIFT[18] and the lab’s SNP database of pre-existing population. ANNOVAR[19]was used to annotate all these SNVs. For somatic mutations, calls were removed if they were present in > 1 % populations in 1,000 Genome Database or in ExAC. The resulting list was further filtered through an in-house mutation list of common sequencing errors. Additionally, a variant with >20% abundance in normal sample, likely an artifact, was also removed from the mutation list. Structural variants were detected using FACTERA with default parameters[20]. and CNVs were detected by ADTEx (GPLv3; <http://adtex.sourceforge.net/>), both with default parameters. The threshold for CNV loss was 0.65 and 2.0 for CNV gain.

**Mutation Description and Statistical Analysis**

Oncoplots, constructed by R (4.0.3), was used to view the overall mutation landscape of ESCC patients in this study. Progression-free survival(PFS) was defined from the date of pathological diagnosis of esophageal carcinoma (EC) to the time of disease progression, worsening, or the last follow-up before progression. Overall survival(OS) started from EC diagnosis to the date of death or last follow-up. Kaplan-Meier method was used to estimate these two outcome measures among different genetic groups, different physiological populations, and selected pathways, followed by a stratified log-rank test for evaluating any differences. Subsequently, univariate Cox hazard models were further performed to define any prognostic factors affecting PFS and OS in this cohort. Statistically significant factors (p-value ≤0.1) defined in the single factor analysis were reviewed in detail. The beta coefficient in the pathway-related univariate analysis was the degree of change in the outcome (PFS or OS) for every 1-unit change in the number of pathway genes expression.

**Results**

**Clinical characteristics and mutation landscape of ESCC patients**

The basic characteristic of 58 enrolled ESCC patients was shown in (Table1). More than half of patients in the cohort were aged above 60 (55.17%), with a median age of 63 years old (range: 41-83 years old). Forty-six patients (79.31%) were male and only 12(20.69%) were female. Around sixty-eight percent (39/58) of the patients were smokers and fifty percentage had history of alcohol consumption(29/58). More than half of the patients were diagnosed with stage III (36/58, 62.07%) ESCC, and 16 people (16/58, 27.59%) had stage II, with additional 6 people (6/58, 10.34%) of stage IV.

In these Asian ESCC patients, *TP53* (54/58, 93.1%) was the most frequent mutation gene, followed by *NOTCH1*(30/58, 51.7%)(Fig 1). Amplification of *MCL1* (24/58, 41.4%), *FGF19* (23/58, 39.7%), *CCND1* (22/58, 37.9%) and *MYC* (20/58, 34.5%) were the four dominant types of CNV identified in this ESCC cohort. As previously mentioned, *FGF19* and *CCND1* were often co-amplified since they were both located at adjacent locations on chromosome 11q13. Interestingly, *YAP1* amplification and *SOX2* amplifications were mutually exclusive to each other in these ESCC patients (Fig 1). A similar negative correlation of protein expression level in *YAP1* and *SOX2* was also found in vivo and in vitro of pancreatic neoplastic cells[21; 22].

**Gene alterations associated with disease outcomes in ESCC patients.**

In this cohort, the *YAP1* variant, *BRIP1* mutant were identified as adverse factors for PFS and OS in univariate analysis. In multivariate analysis, *YAP1* variant, *BRIP1* mutant significantly associated with OS but not with PFS (Table 2). Kaplan-Meier plot revealed that median PFS (mPFS) and median OS (mOS) of patients with *YAP1* variant was 8.61 months and 12.55 months respectively, which were significantly shorter than that of *YAP1* wild type patients (Figure 2A-B). ESCC Patients with *BRIP1* mutant also displayed worse outcomes compared to ones with *BRIP1* wild type, achieving an mPFS of 5.87 months and an mOS of 11.38 months (Figure 2C-D). *SOX2* amplification, which was mutually exclusive to *YAP1* in this cohort, did not reach statistical significance in univariant analysis (Figure 2E-F).

**Prognosis value of cancer-associated pathways in ESCC**

Pathway analysis was performed according to the genes in ten cancer-associated pathways in literature(Table 3)[9]. The individual genes in each pathway included were listed in Table S1. Around 93% of this EC cohort harbored TP53 signaling pathway alterations. Altered NOTCH (72.41%), RTK-RAS (68.97%), and Cell cycle (53.45%) pathway genes were identified in more than 50% of the total cases (Figure S2 &Table S3). EC Patients with mutations in *Keap1*-*Nrf2* pathway had much shorter (n = 2, mPFS = 2.75, beta = 3.48, p < 0.0001, HR (95%CI =32.5(4.48-235)) PFS than wild-type patients (n = 56, mPFS = 16.2) (Fig 3A). Similarly, mutations in this pathway also increased the risk of unfavourable OS (n = 2, beta coefficient = 29, p < 0.0001) than the wild type counterpart (n = 56, mOS = 26.0)(Fig 3B). We also validated this observation using an independent cohort of 88 ESCC patients with OS information[23]. As shown in Figure S3, seven patients had altered *Keap1*-*Nrf2* pathway with a significantly shortened OS compared to patients with the intact *Keap1*-*Nrf2* pathway (p=0.039).

In contrast to *Keap1*-*Nrf2* pathway aberrations, patients with mutations in the *PI3K*-*Akt* pathway displayed a longer PFS (n = 26, mPFS = 22, beta = 0.74, p = 0.0337, HR (95% CI) = 0.48(0.24-0.96)) and longer OS (n = 26, mOS = 34.69, beta = 0.71, p = 0.0495, HR (95% CI) = 32.5(0.24-1.01). Comparatively, wild type patients achieved a shorter PFS (n = 32, mPFS = 9.8) and OS (n = 32, mOS = 17.68) (Fig 3C-D). [24]In patients with *PI3K-Akt* pathway alterations, three were found with altered *PTEN* and seven were found with altered *PIK3CA*. Patients with *PIK3CA* mutation tend to have longer PFS and OS than patients with wild type *PIK3CA.* The altered *PTEN* did not show association with PFS or OS in this cohort(Figure S4).

A representative case of an ESCC patient with *NFE2L2* mutation was shown in Figure 3E. The patient was a 49-year-old male diagnosed with Stage IV ESCC. He was identified with *NFE2L2* D29G mutation at an allele frequency (AF) of 48.19% before treatment. *RB1* frameshift mutation and *TP53* G262V were identified at an AF of 52.13% and 37.25% respectively at the same time. The tumor quickly progressed after 2.89 months of dCRT and metastasized to distant lymph nodes. Eventually, the patient died after 5.91months of chemoradiotherapy and chemotherapy.

**Discussion**

In this study, we retrospectively studied the clinical features and cancer genomes of 58 patients with inoperable ESCC tumors, intending to identify prognostic biomarkers for Chinese ESCC patients. Among all the baseline clinical characteristics, gender appeared to be an independent prognostic factor, which was in accord with the previous study[25]. The high frequency of gene amplification was another genetic feature observed in esophageal squamous cell carcinoma. In our cohort, 75.9% (44/58) patients had at least one gene amplified. *MCL1* (24/58, 41.4%), *FGF19* (22/58, 37.9%), *CCND1* (22/58, 37.9%) and *MYC* (20/58, 34.5%) were the four dominant amplified genes. Besides, we found that *YAP1* and *SOX2* were exclusively amplified in different patients in this cohort. By further reviewing the prognosis of patients with/without *YAP1* and *SOX2* amplification, patients without double amplification were found to have the best PFS and OS. The group of patients with *SOX2* amplification and the group with *YAP1* amplification both obtained shorter PFS and OS, which was associated with the previous study[11].

Interestingly, the exclusion of *YAP1* amplification and *SOX2* amplification was only reported in one mouse model study, that Yap loss intended to induce acute metabolic stress, leading to epigenetic reprogramming with *SOX2* upregulation[21]. Most other studies showed that *YAP1* is co-amplified with *SOX2* by *YAP1* binding to *SOX2*‘s enhancer region, and *SOX2* may in-turn restore *YAP1* through antagonizing the Hippo pathway in maintaining cell stemness and leading to poor prognosis. The cooperation of *YAP1* and *SOX2* was detected in various cancer types, including osteosarcoma, urothelial cancer, and HNSCC (head and neck squamous cell carcinoma)[21; 26]. Thus, behind the scenes of mutual exclusion for *SOX2* amplification and *YAP1* amplification of these patients in this study, there lies a unique unknown molecular mechanism of ESCC tumorigenesis，distinguished from other cancer types, that needs further investigation.

Of the two pathways identified as potential prognostic biomarkers of ESCC, the *Keap1*-*Nrf2* pathway is known for inducing chemoradioresistance[27; 28]. One of the major roles in *Nrf 2* is to initiate cytoprotective responses under oxidant stress by binding to and activating antioxidant response element (ARE) in the modular regions of its downstream targets[29]. In addition, *Nrf2* promotes cell proliferation and metabolic reformation by triggering metabolic genes. On the other hand, *Keap1* can inhibit *Keap1*-*Nrf2* pathway by suppressing the expression of *Nrf2*. Under oxidative stress and electrophilic stress, the confirmation of *Keap1* is reconstructed due to alterations in its cysteine residues. Newly synthesized *Nrf2* can bypass *Keap1* and translocate into the nucleus by *Keap1* proteins inactivation or *Keap1*-*Nrf2* complex disrupting[29]. Here, the two patients carrying mutations in *Keap1*-*Nrf2* pathway both exhibit poor disease outcomes with shorter PFS and OS compared to *Keap1*-*Nrf2* pathway wild-type patients. The rapid progression of patients carrying abnormalities in *Keap1*-*Nrf2* pathway in other cancer types was reported in several studies[30; 31]. Due to the limited number of patients with altered *Keap1*-*Nrf2* pathway in this study, further research was needed to identify whether activating mutations of *Keap1*-*Nrf2* pathway is a potential chemo-radioresistant-related biomarker for patients receiving dCRT therapy.

*PIK3CA* mutation was a commonly reported factor for treatment and prognosis in ESCC patients, but conflicting conclusions were drawn across studies[24; 32; 33]. Our studies showed a favorable prognosis among the patients with muted *PI3K* pathway. PI3K-AKT pathway is considered one of the master regulators for cancer and ideal targets for anti-cancer drugs[34]. It is known to play an important role in the development and progression of many solid cancers[35; 36; 37]. Further in-vivo study or expansion of cohort size was needed to confirm our results.

**Ethical approval and consent to participate**

Patient consent form was obtained from each patient following the guideline of Institutional Review Board requirements and the Declaration of Helsinki

**Consent for publication**

No individual data were used in this study.

**Availability of supporting data**

All data that support the findings of this study are available from the corresponding authors upon a reasonable request.

**Competing interests**

Ruoying Yu, Jingwen Liu, Jiaohui Pang, Junli Zhang, Xue Wu, and Yang Shao are shareholders or employees of Nanjing Geneseeq Technology Inc.  The remaining authors have no conflicts of interest to declare.

**Funding**

This study was supported in part by the Natural Science Foundation of Shandong (Grant No. ZR2020MH229), as well as the special foundation for CSCO Cancer Research (Grant No. Y-QL2019-0149 and Y-2019AZMS-0522) and the project of Shandong University (Grant No. 199/2019 heng).

**Authors’ contribution**

Study design: Qiang Li, Zhe Yang, Honghai Dai

Data acquisition: Honghai Dai, Yanjun Wei, Yunxia Liu

Data analysis: Ruoying Yu, Jingwen Liu, Jiaohui Pang, Junli Zhang

Manuscript writing: Honghai Dai, Yanjun Wei, Ruoying Yu, Jingwen Liu, Jiaohui Pang, Junli Zhang

Study supervision: Xue Wu, Yang Shao, Qiang Li, Zhe Yang, Honghai Dai

**Acknowledgements**

We would like to thank the patients who participated in this study and their family, as well as the investigators and research staff involved.

References

[1] F. Bray, J. Ferlay, I. Soerjomataram, R.L. Siegel, L.A. Torre, and A. Jemal, Global cancer statistics 2018: GLOBOCAN estimates of incidence and mortality worldwide for 36 cancers in 185 countries. CA: a cancer journal for clinicians 68 (2018) 394-424.

[2] C.C. Abnet, M. Arnold, and W.Q. Wei, Epidemiology of Esophageal Squamous Cell Carcinoma. Gastroenterology 154 (2018) 360-373.

[3] J. Dent, H.B. El-Serag, M.A. Wallander, and S. Johansson, Epidemiology of gastro-oesophageal reflux disease: a systematic review. Gut 54 (2005) 710-7.

[4] P.H. Viale, The American Cancer Society's Facts & Figures: 2020 Edition. Journal of the advanced practitioner in oncology 11 (2020) 135-136.

[5] F. Tustumi, C.M. Kimura, F.R. Takeda, R.H. Uema, R.A. Salum, U. Ribeiro-Junior, and I. Cecconello, Prognostic Factors and Survival Analysis in Esophageal Carcinoma. Arq Bras Cir Dig 29 (2016) 138-141.

[6] Y.M. Yang, P. Hong, W.W. Xu, Q.Y. He, and B. Li, Advances in targeted therapy for esophageal cancer. Signal Transduct Target Ther 5 (2020) 229.

[7] M. Takahashi, K. Kato, M. Okada, K. Chin, S. Kadowaki, Y. Hamamoto, Y. Doki, Y. Kubota, H. Kawakami, T. Ogata, H. Hara, M. Muto, Y. Nakashima, R. Ishihara, M. Tsuda, S. Motoyama, M. Kodani, and Y. Kitagawa, Nivolumab versus chemotherapy in Japanese patients with advanced esophageal squamous cell carcinoma: a subgroup analysis of a multicenter, randomized, open-label, phase 3 trial (ATTRACTION-3). Esophagus 18 (2021) 90-99.

[8] J.H. Choe, S. Mazambani, T.H. Kim, and J.W. Kim, Oxidative Stress and the Intersection of Oncogenic Signaling and Metabolism in Squamous Cell Carcinomas. Cells 10 (2021).

[9] F. Sanchez-Vega, M. Mina, J. Armenia, W.K. Chatila, A. Luna, K.C. La, S. Dimitriadoy, D.L. Liu, H.S. Kantheti, S. Saghafinia, D. Chakravarty, F. Daian, Q. Gao, M.H. Bailey, W.W. Liang, S.M. Foltz, I. Shmulevich, L. Ding, Z. Heins, A. Ochoa, B. Gross, J. Gao, H. Zhang, R. Kundra, C. Kandoth, I. Bahceci, L. Dervishi, U. Dogrusoz, W. Zhou, H. Shen, P.W. Laird, G.P. Way, C.S. Greene, H. Liang, Y. Xiao, C. Wang, A. Iavarone, A.H. Berger, T.G. Bivona, A.J. Lazar, G.D. Hammer, T. Giordano, L.N. Kwong, G. McArthur, C. Huang, A.D. Tward, M.J. Frederick, F. McCormick, M. Meyerson, N. Cancer Genome Atlas Research, E.M. Van Allen, A.D. Cherniack, G. Ciriello, C. Sander, and N. Schultz, Oncogenic Signaling Pathways in The Cancer Genome Atlas. Cell 173 (2018) 321-337 e10.

[10] W. Fang, Y. Ma, J.C. Yin, S. Hong, H. Zhou, A. Wang, F. Wang, H. Bao, X. Wu, Y. Yang, Y. Huang, H. Zhao, Y.W. Shao, and L. Zhang, Comprehensive Genomic Profiling Identifies Novel Genetic Predictors of Response to Anti-PD-(L)1 Therapies in Non-Small Cell Lung Cancer. Clinical cancer research : an official journal of the American Association for Cancer Research 25 (2019) 5015-5026.

[11] H. Dai, Y.W. Shao, X. Tong, X. Wu, J. Pang, A. Feng, and Z. Yang, YAP1 amplification as a prognostic factor of definitive chemoradiotherapy in nonsurgical esophageal squamous cell carcinoma. Cancer medicine 9 (2020) 1628-1637.

[12] D.C. Koboldt, Q. Zhang, D.E. Larson, D. Shen, M.D. McLellan, L. Lin, C.A. Miller, E.R. Mardis, L. Ding, and R.K. Wilson, VarScan 2: somatic mutation and copy number alteration discovery in cancer by exome sequencing. Genome research 22 (2012) 568-76.

[13] S.T. Sherry, M.H. Ward, M. Kholodov, J. Baker, L. Phan, E.M. Smigielski, and K. Sirotkin, dbSNP: the NCBI database of genetic variation. Nucleic acids research 29 (2001) 308-11.

[14] M.J. Landrum, J.M. Lee, M. Benson, G. Brown, C. Chao, S. Chitipiralla, B. Gu, J. Hart, D. Hoffman, J. Hoover, W. Jang, K. Katz, M. Ovetsky, G. Riley, A. Sethi, R. Tully, R. Villamarin-Salomon, W. Rubinstein, and D.R. Maglott, ClinVar: public archive of interpretations of clinically relevant variants. Nucleic acids research 44 (2016) D862-8.

[15] C. Genomes Project, A. Auton, L.D. Brooks, R.M. Durbin, E.P. Garrison, H.M. Kang, J.O. Korbel, J.L. Marchini, S. McCarthy, G.A. McVean, and G.R. Abecasis, A global reference for human genetic variation. Nature 526 (2015) 68-74.

[16] K.J. Karczewski, B. Weisburd, B. Thomas, M. Solomonson, D.M. Ruderfer, D. Kavanagh, T. Hamamsy, M. Lek, K.E. Samocha, B.B. Cummings, D. Birnbaum, C. The Exome Aggregation, M.J. Daly, and D.G. MacArthur, The ExAC browser: displaying reference data information from over 60 000 exomes. Nucleic acids research 45 (2017) D840-D845.

[17] S.A. Forbes, D. Beare, P. Gunasekaran, K. Leung, N. Bindal, H. Boutselakis, M. Ding, S. Bamford, C. Cole, S. Ward, C.Y. Kok, M. Jia, T. De, J.W. Teague, M.R. Stratton, U. McDermott, and P.J. Campbell, COSMIC: exploring the world's knowledge of somatic mutations in human cancer. Nucleic acids research 43 (2015) D805-11.

[18] P.C. Ng, and S. Henikoff, SIFT: Predicting amino acid changes that affect protein function. Nucleic acids research 31 (2003) 3812-4.

[19] K. Wang, M. Li, and H. Hakonarson, ANNOVAR: functional annotation of genetic variants from high-throughput sequencing data. Nucleic acids research 38 (2010) e164.

[20] A.M. Newman, S.V. Bratman, H. Stehr, L.J. Lee, C.L. Liu, M. Diehn, and A.A. Alizadeh, FACTERA: a practical method for the discovery of genomic rearrangements at breakpoint resolution. Bioinformatics 30 (2014) 3390-3.

[21] S. Murakami, I. Nemazanyy, S.M. White, H. Chen, C.D.K. Nguyen, G.T. Graham, D. Saur, M. Pende, and C. Yi, A Yap-Myc-Sox2-p53 Regulatory Network Dictates Metabolic Homeostasis and Differentiation in Kras-Driven Pancreatic Ductal Adenocarcinomas. Dev Cell 51 (2019) 113-128 e9.

[22] E. Seo, U. Basu-Roy, P.H. Gunaratne, C. Coarfa, D.S. Lim, C. Basilico, and A. Mansukhani, SOX2 regulates YAP1 to maintain stemness and determine cell fate in the osteo-adipo lineage. Cell reports 3 (2013) 2075-87.

[23] Y. Song, L. Li, Y. Ou, Z. Gao, E. Li, X. Li, W. Zhang, J. Wang, L. Xu, Y. Zhou, X. Ma, L. Liu, Z. Zhao, X. Huang, J. Fan, L. Dong, G. Chen, L. Ma, J. Yang, L. Chen, M. He, M. Li, X. Zhuang, K. Huang, K. Qiu, G. Yin, G. Guo, Q. Feng, P. Chen, Z. Wu, J. Wu, L. Ma, J. Zhao, L. Luo, M. Fu, B. Xu, B. Chen, Y. Li, T. Tong, M. Wang, Z. Liu, D. Lin, X. Zhang, H. Yang, J. Wang, and Q. Zhan, Identification of genomic alterations in oesophageal squamous cell cancer. Nature 509 (2014) 91-5.

[24] H. Shigaki, Y. Baba, M. Watanabe, A. Murata, T. Ishimoto, M. Iwatsuki, S. Iwagami, K. Nosho, and H. Baba, PIK3CA mutation is associated with a favorable prognosis among patients with curatively resected esophageal squamous cell carcinoma. Clinical cancer research : an official journal of the American Association for Cancer Research 19 (2013) 2451-9.

[25] N. Pandeya, C.M. Olsen, and D.C. Whiteman, Sex differences in the proportion of esophageal squamous cell carcinoma cases attributable to tobacco smoking and alcohol consumption. Cancer Epidemiol 37 (2013) 579-84.

[26] H. Omori, K. Sato, T. Nakano, T. Wakasaki, S. Toh, K. Taguchi, T. Nakagawa, and M. Masuda, Stress-triggered YAP1/SOX2 activation transcriptionally reprograms head and neck squamous cell carcinoma for the acquisition of stemness. Journal of cancer research and clinical oncology 145 (2019) 2433-2444.

[27] J. Zhang, Q. Jiao, L. Kong, J. Yu, A. Fang, M. Li, and J. Yu, Nrf2 and Keap1 abnormalities in esophageal squamous cell carcinoma and association with the effect of chemoradiotherapy. Thoracic cancer 9 (2018) 726-735.

[28] K. Taguchi, and M. Yamamoto, The KEAP1-NRF2 System in Cancer. Frontiers in oncology 7 (2017) 85.

[29] E. Kansanen, S.M. Kuosmanen, H. Leinonen, and A.L. Levonen, The Keap1-Nrf2 pathway: Mechanisms of activation and dysregulation in cancer. Redox Biol 1 (2013) 45-9.

[30] F. Goeman, F. De Nicola, S. Scalera, F. Sperati, E. Gallo, L. Ciuffreda, M. Pallocca, L. Pizzuti, E. Krasniqi, G. Barchiesi, P. Vici, M. Barba, S. Buglioni, B. Casini, P. Visca, E. Pescarmona, M. Mazzotta, R. De Maria, M. Fanciulli, G. Ciliberto, and M. Maugeri-Sacca, Mutations in the KEAP1-NFE2L2 Pathway Define a Molecular Subset of Rapidly Progressing Lung Adenocarcinoma. Journal of thoracic oncology : official publication of the International Association for the Study of Lung Cancer 14 (2019) 1924-1934.

[31] C. Zoja, A. Benigni, and G. Remuzzi, The Nrf2 pathway in the progression of renal disease. Nephrol Dial Transplant 29 Suppl 1 (2014) i19-i24.

[32] S. Wada, T. Noguchi, S. Takeno, and K. Kawahara, PIK3CA and TFRC located in 3q are new prognostic factors in esophageal squamous cell carcinoma. Annals of surgical oncology 13 (2006) 961-6.

[33] L. Wang, L. Shan, S. Zhang, J. Ying, L. Xue, Y. Yuan, Y. Xie, and N. Lu, PIK3CA gene mutations and overexpression: implications for prognostic biomarker and therapeutic target in Chinese esophageal squamous cell carcinoma. PloS one 9 (2014) e103021.

[34] J. Yang, J. Nie, X. Ma, Y. Wei, Y. Peng, and X. Wei, Targeting PI3K in cancer: mechanisms and advances in clinical trials. Molecular cancer 18 (2019) 26.

[35] L. Song, H. Xiong, J. Li, W. Liao, L. Wang, J. Wu, and M. Li, Sphingosine kinase-1 enhances resistance to apoptosis through activation of PI3K/Akt/NF-kappaB pathway in human non-small cell lung cancer. Clinical cancer research : an official journal of the American Association for Cancer Research 17 (2011) 1839-49.

[36] M. Jiao, and K.J. Nan, Activation of PI3 kinase/Akt/HIF-1alpha pathway contributes to hypoxia-induced epithelial-mesenchymal transition and chemoresistance in hepatocellular carcinoma. International journal of oncology 40 (2012) 461-8.

[37] L.C. Vredeveld, P.A. Possik, M.A. Smit, K. Meissl, C. Michaloglou, H.M. Horlings, A. Ajouaou, P.C. Kortman, D. Dankort, M. McMahon, W.J. Mooi, and D.S. Peeper, Abrogation of BRAFV600E-induced senescence by PI3K pathway activation contributes to melanomagenesis. Genes & development 26 (2012) 1055-69.

**Figure Legend**

**Fig 1. The mutational pattern in Chinese ESCC patients**

The upper oncoplot showed the mutational landscape of patients in this cohort.

The lower oncoplot showed that YAP1 gain and SOX2 gain were mutually exclusive to each other. No patient had both amplification at the same time.

**Fig 2 Survival analysis of ESCC patients with YAP1 mutation, BRIP1 variation, and SOX2 mutation.**

A. Kaplan-Meier plot showed PFS of the subgroup patients with YAP1 mutation versus patients without YAP1 mutation.

B. Kaplan-Meier plot showed OS of the subgroup patients with YAP1 mutation versus patients without YAP1 mutation.

C. Kaplan-Meier plot showed PFS of the subgroup patients with BRIP1 mutation versus patients without BRIP1 variation.

D. Kaplan-Meier plot showed OS of the subgroup patients with BRIP1 mutation versus patients without BRIP1 variation.

E. Kaplan-Meier plot showed PFS of the subgroup patients with SOX2 amplification versus patients without SOX2 mutation.

F. Kaplan-Meier plot showed OS of the subgroup patients with SOX2 amplification versus patients without SOX2 mutation.

**Fig 3 Survival analysis of ESCC patients with altered oncogenic pathways**

A. Kaplan-Meier plot for PFS of ESCC patients with intact or altered Keap1-Nrf2 pathway.

B. Kaplan-Meier plot for OS of ESCC patients with intact or altered Keap1-Nrf2 pathway.

C. Kaplan-Meier plot for PFS of ESCC patients with intact or altered PI3K-Akt pathway.

D. Kaplan-Meier plot for OS of ESCC patients with intact or altered PI3K-Akt pathway.

E. Representative case of a patient with Keap1-Nrf2 pathway alterations.
